# Generation Z Young People’s Perception of Sexist Female Stereotypes about the Product Advertising in the Food Industry: Influence on Their Purchase Intention

**DOI:** 10.3390/foods11010053

**Published:** 2021-12-27

**Authors:** Guillermo Bermúdez-González, Eva María Sánchez-Teba, María Dolores Benítez-Márquez, Amanda Montiel-Chamizo

**Affiliations:** 1 Department of Business Management, Faculty of Commerce and Management, University of Malaga, 29071 Malaga, Spain; gjbermudez@uma.es (G.B.-G.); 0619693781@alu.uma.es (A.M.-C.); 2 Department of Business Management, Faculty of Economics and Business, University of Malaga, 29070 Malaga, Spain; emsanchezteba@uma.es; 3 Department of Applied Economics (Statistics and Econometrics), Faculty of Economics and Business, University of Malaga, 29070 Malaga, Spain

**Keywords:** Generation Z, advertising, gender stereotypes, ambivalent sexism, ethical judgment, attitude towards advertising, purchase intention, structural equation modeling, PLS-SEM

## Abstract

Previous studies have generated important insights into consumer behavior. However, no study has addressed how to persuade young people belonging to Generation Z to increase the purchase intention of food products from a gender perspective. Drawing on ambivalent sexism theory, this paper explores the influence of the attitude toward advertising and the ethical judgment to predict consumers’ food product purchase intention. We applied a quantitative method, partial least squares structural equation modeling, to 105 individuals. Two advertisements with different food products and female role stereotype categories are using: (1) women in a traditional role or housewife’s role (benevolent sexism), and (2) women in a decorative role or physical attractiveness (hostile). However, the results show that attitude toward advertising has a direct and positive influence on purchase intention in advertisement with benevolent sexism. In addition, the effect of ethical judgment on consumers’ food product purchase intention is not significant. In the advertisement with hostile sexism, both—attitude toward advertising and ethical judgment—directly and positively impact purchase intention. The study provides a novelty conceptual model in the food industry for Generation Z and recommendations on the use of female sexist stereotypes in food and beverage advertising.

## 1. Introduction

Gender stereotypes are defined as general beliefs about the roles, behaviors, and psychological characteristics that describe women and men [1]. Sexualizing women in the media promotes sexism and female stereotypes, making it essential to study it. Different authors have considered these gender identities socially constructed, and advertising proposes individuals use lifestyles to define their role in society, prescribing desired relationships and behaviors for women and men [2,3].

As Furnham and Li [4] indicate, food advertisements are sometimes perceived as the instrument on which some brands and industries capitalize on female stereotypes, especially in terms of image, family care, and health. The nutritional focus of many food products is perceived as a “feminine” concern, even if the product category does not have a particular gender bias. Gender theory has facilitated debate on the social and ethical implications of advertising associated with the communication of female role stereotypes. This theory provides a basis for academic marketing to explore how advertising accurately reflects women, rather than stereotypically representing them [5,6]. From the perspective of this general theory, different authors have approached the issue from the so-called ambivalent sexism theory. From this point of view, sexism is multidimensional, encompassing notions of hostile and benevolent sexism that manifest themselves in categories of female role stereotypes in advertising [7].

Research on the use of sexist female role types and their effectiveness in traditional and digital media has been analyzed by the scientific community [6,8,9,10]. The principal roles detected are male dependency and women as homemakers (traditional roles), women who must take care of their physical appearance, and women as sex objects (decorative roles). Even though other egalitarian and women-empowering roles are increasing in communication strategies, sexist roles are still present in advertising [11] and influence purchase intention [12]. However, academia has not delved into young people’s perception of such advertising stereotypes and the influence of this perception on the purchase intention of the advertised food products. Moreover, the few existing studies are very descriptive and reflect the perception of previous generations [13].

The present research focuses on Generation Z, the group of young people born between 1994 and 2010 [14,15], by jointly analyzing the effect on the intention to purchase food products of two perceptual dimensions that have never been used together in the theory of ambivalent sexism, attitude toward the advertisement and ethical judgment, to fill the gap above-mentioned. The originality of the article lies not only in the scope of the study and the target group; but also in the fact that it is a novelty to jointly analyze the effect of the dimensions on this generation’s intention to purchase food products and their attitude toward advertising’s mediating impact.

## 2. Literature Review and Hypotheses

### 2.1. Generation Z

The different authors who have tried to conceptualize generational theory contradict the time interval that makes up this cohort generation encompasses those born in a period of approximately 20 years [16] who must meet three membership requirements: feeling part of the generational group; sharing behavior and beliefs, and having ordinary experiences in their childhood and adolescence [17]. This empirical study references the section born between 1994 and 2010 [14]. Opinions and perceptions of each generation are crucial to know how to treat and what to offer each segment [18] and to understand their effect on purchase intention.

This generation presents itself as the great challenge for marketing, as it will be the engine of consumption, innovation, and change, so it is relevant to explore the expectations and perceptions of this generation with greater power than the previous ones to redefine production and consumption in retail [19]. Moreover, Generation Z accounts for more than 30% of the world’s population [20] and makes up a quarter of the UK population, with high purchasing power, and almost 40% of all U.S. consumers [19]. They spend a lot of time and effort in the buying process trying to justify their purchases as intelligent, although the influence of advertisement and the opinions of others through networks and interpersonal communication are relevant [21]. This generation decides what is captivating, and if they do not like the media, they create their channels [22]. It is crucial for different sectors, including the food distribution sector, to study how advertising influences purchase intention. This generation is deeply marked by audio-visual culture, especially digital culture, integrating new technologies into their personal and professional lives, and social networks [23]. Other studies have considered these young people to be more tolerant of cultural diversity and are concerned about race, class, and gender inequalities [24]. In addition, they have fewer social skills and potential problems working under pressure [25]. However, the influence of sexist stereotypes in advertising on purchase intention is unknown in this generation. Similarly, the academy did not study in depth whether this influence is direct or inverse, depending on the category of sexism present in the advertisements. This fact justifies the purpose of this study.

### 2.2. Perception of Gender Stereotypes in Food and Drink Advertising

McArthur and Resko’s study published in the last quarter of the 20th century was the pioneering study on gender role stereotypes in television advertising in the United States [26]. There is comprehensive and growing literature on gender stereotypes and the representation of these roles in advertising [4,27,28,29]. These studies have been organized in different media such as print and radio [30], magazines [31], television [32], and social networks or websites [6,33].

According to Browne [34], gender stereotypes are general beliefs about the traits and roles, psychological characteristics, and behaviors that describe women and men. Gender theory has facilitated the debate on the social and ethical implications of advertising associated with the communication of female role stereotypes [6]. At the end of the 20th century, different studies confirmed that advertising in popular media is a primary way to introduce female stereotypes and promote desired behaviors, making its systematic study essential [35]. Since then, academic research has been concerned with carefully exploring how advertising accurately reflects females rather than portraying them in stereotypical and sexist ways. Furthermore, sexism has become a multidimensional construct encompassing the notions of hostile and benevolent sexism (ambivalent sexism theory), which manifest themselves in different categories of female role stereotypes in advertising. The first form of sexism, hostile sexism, characterizes women as incapable of making important decisions and portrays women as easily manipulated, vulnerable, and weak. In this typology, criticism is unpleasant, obstructive, and directed at women who do not conform to traditional role models. The second form of sexism, benevolent sexism, is subtler and affectionate toward women while showing their inferiority and male dependence. This form of sexism promotes traditional female roles and pretends to depict women concerned with their physical attractiveness in advertising [36].

Following Plakoyiannaki et al. [6], we can classify female role stereotypes into four broader categories: women in traditional roles (dependents and homemakers); decorative women (related to physical attractiveness and as sex objects); women in non-traditional roles (non-traditional activities, career-oriented women with authority and charisma); and women in neutral roles (represented as equal to men). The first two role categories (traditional and decorative) can be associated with sexism (benevolent and hostile), as opposed to the latter two (non-sexist). This classification of female stereotypes has a relation to previous categorizations of images of women in advertising [6,37].

Our research focuses exclusively on the perception of sexist roles in two-mentioned categories: The women’s role concerned with physical attractiveness (category women in the decorative roles) and the women roles of a housewife (category women in the traditional roles).

Focusing on food and drink advertising, some authors have claimed that female stereotypes are recurrent in traditional and decorative roles, especially concerning products linked to body image, health, or body care [4]. The nutritional or aesthetic focus of many food products is perceived as a female concern, even if the product category as a whole has no particular gender bias and endows food with an emotional bias when it comes to women [38].

Others researchers [39] have considered that advertising is particularly sexist in the housewife role and specifically in the activity of feeding the family. While it is true that this task avoided being directly attributed to women, observing that either she is the one who mainly supervises the activity of the characters is diluted in the background to avoid making the conflictive nature of domestic work visible. However, Zawisza et al. [12], in their study in different countries, showed that the presence of benevolent sexist stereotypes positively influences intention. However, the perception of hostile sexism in advertising influenced product purchase intention negatively in some countries, but positively in others.

Although there have been no specific studies on young people’s perception of gender roles in food products, researchers have analyzed the presence or absence of these stereotypes. Thus, Aronovsky and Furnham [40] conducted a study of gender stereotypes in advertising for 153 food products on British television. Although both sexes illustrated stereotypical ways, late-night advertisements revealed a higher proportion of sexist female portrayals. Additionally, in the context of adverts, the importance of advertising language, the impact of food and drink advertising [41], its influence on purchase intention and brand loyalty [42] have been investigated. In this sense, around 35% of advertisements aimed at children and young people are for food and drink, following [43].

### 2.3. The Influence of Ethical Judgment on the Intention to Purchase the Product and the Effect of Attitude toward Sexist Advertising on the Intention to Purchase the Product

#### 2.3.1. Direct and Indirect Influence of Ethical Judgment toward Sexist Advertising on Purchase Intention

The purchase intention is the result of the expected behavior of consumers toward the purchase or not of a product, and can be affected by a multitude of factors [44], business ethics being one of them [45]. Some academics consider ethical values as a determinant in the purchase of food (e.g., [46]). Consumers are increasingly demanding the ethical behavior of companies in the food sector [47], judging and evaluating, among others, the communication actions carried out by companies.

Brito-Rhor et al. [11] define ethical judgment as the power to reason which conduct or action is the most appropriate, from among a set of alternatives, based on the values of the society where we live [11]. Outside this industry, some researchers have analyzed the influence of ethical judgment on purchase intention, concluding a direct and positive effect [48,49,50,51]. Additionally, other academics have concluded that the stereotypical portrait of women was much more present at the beginning of this century than at the end of the previous one [52,53]. There have been no specific studies on the indirect relationship between ethical judgment and purchase intention, thanks to the mediating power of attitude toward advertising. However, Simpson et al. [49] considered both constructs as antecedents of the purchase intention.

In the food sector, existing studies analyze how different aspects of advertising ethics influence purchase intention. Thus, some have focused on fraudulent product information on labeling [54], others on misleading advertising [55], or the sexualization of advertising [56]. For their part, Hernandez and Kaeck [47] concluded that there was a direct influence of ethical judgment on the intention to purchase the advertised food or drink. Moreover, other works have shown that inappropriate ethical behavior by the advertiser will have negative repercussions on the intention to purchase food products [57,58]. Nevertheless, to our best knowledge, the relationship between ethical judgment and purchase intention has not been tested yet from the perception of young people of Generation Z.

Keeping in mind all of these considerations, we postulate the following hypotheses:

**Hypothesis 1** **(1).** *Ethical judgment has a direct and positive influence on purchase intention in video 1.*

**Hypothesis 1** **(2).** *Ethical judgment has a direct and positive influence on purchase intention in video 2.*

#### 2.3.2. Influence of the Attitude toward Sexist Advertising on the Purchase Intention

The attitude associated with advertising perception has been conceptualized by the scientific literature mainly with the terms “ad attitude” [59,60] and “attitude toward” [61,62]. Lee at al. [63] defined this ad attitude as the favorable or unfavorable responses to a particular advertisement that may lead to an emotional change. Moreover, the theory of planned behavior considers attitudes as the key drivers of behavior intentions [64]. Thus, the purchasing decision process of consumers will be affected by attitudes [60], and different perceptions of advertising will lead to different affective responses from consumers [34].

Multiple studies have demonstrated the relationship between attitude toward advertising and purchase intention [34,51,65,66]. In some cases, this relationship has been analyzed from specific study areas such as mobile advertising, smartphone advertising [63,67], controversial advertisements [68], celebrity-product match up on Facebook [69], or in sexist advertising [70]. However, to our best knowledge, the influence of attitude toward advertisements on purchase intention has not been analyzed in the specific field of food product advertising or from the perspective of Generation Z.

Based on the aforementioned literature, we propose the following hypothesis:

**Hypothesis 2** **(1).** *Attitude toward advertisement has a direct, positive influence on purchase intention in video 1.*

**Hypothesis 2** **(2).** *Attitude toward advertisement has a direct, positive influence on purchase intention in video 2.*

We also postulate the following hypothesis:

**Hypothesis 3** **(1).** *Attitude toward advertisement mediates between ethical judgment and purchase intention in video 1.*

**Hypothesis 3** **(2).** *Attitude toward advertisement mediates between ethical judgment and purchase intention in video 2.*

#### 2.3.3. Influence of Ethical Judgment on the Attitude toward Sexist Advertising

If an advertising message is considered ethically correct, positive responses are generated such as commitment to the product and appreciation of the brand. On the other hand, messages are perceived as amoral elicit negative responses [71]. More recent studies [11,56] have demonstrated the direct and positive effect of ethical judgment on the attitude toward advertising. Furthermore, to our best knowledge, there is no evidence of the relationship between ethical judgment and the attitude toward advertising in the food sector concerning young people belonging to Generation Z.

Therefore, we developed the following hypothesis based on the literature review:

**Hypothesis 4** **(1).** *Ethical judgment has a direct, positive influence on attitude toward advertisement in video 1.*

**Hypothesis 4** **(2).** *Ethical judgment has a direct, positive influence on attitude toward advertisement in video 2.*

Other studies have studied the moderated role of other variables such as celebrity-product match-up between attitude toward advertisement and purchase intention [69]. However, we did not find any study in the literature that considered the variable gender as a moderator variable between attitude toward advertisement and purchase intention. In addition, we did not find any research considering gender’s mediation role between ethical judgement and purchase intention. Thus, we established the following hypotheses:

**Hypothesis 5** **(1).** *Gender moderates the relationship between attitude toward advertisement and purchase intention in video 1.*

**Hypothesis 5** **(2).** *Gender moderates the relationship between attitude toward advertisement and purchase intention in video 2.*

**Hypothesis 6** **(1).** *Gender moderates the relationship between ethical judgement and purchase intention in video 1.*

**Hypothesis 6** **(2).** *Gender moderates the relationship between ethical judgement and purchase intention in video 2.*

Finally, we engaged in exploring whether the hypotheses established previously—H1 to H6—are supported in both models (benevolent and hostile sexism).

**Hypothesis** **7.** *The benevolent sexism model and the hostile sexism model are different.*

## 3. Data Collection and Method

### 3.1. Sample and Data Collection

We used two preliminary questionnaires following Churchill’s advice [72]. The former questionnaire was answered through ten interviews with advertising agency directors/managers to detect comprehension differences. Afterward, this first draft was revised by seven experts including university lecturers and professionals. The final version was designed based on their suggestions to improve the deficiencies to have a better understanding. The selection of participants was made using a convenience sampling among young people aged 18–26 years belonging to Generation Z. They were informed that the purpose was to conduct a survey and that their responses would be used in scientific research. The questionnaire was distributed online by Google Forms to university students. The cross-sectional data collection was conducted from December 2020 to April 2021.

To estimate the minimum required sample size, the free program G*Power (version 3.1.9.2, Heinrich-Heine-Universität Düsseldorf, Düsseldorf, Germany) was used [73,74]. According to the configuration of medium effect size (f^2^), the highest number of predictors of a dependent construct is two, a power of 0.80 and a significance of 0.05; the calculated minimum sample should be 68 cases [75] p. 75. However, the questionnaire was completed by 137 participants. Data were filtered (outlier, incongruent responses). The final sample size was composed of 105 valid questionnaires, 65 females and 40 males.

Two recent videos (2014 and 2015) represent the categories of advertising female sexist roles, according to the classification by Plakoyiannaki et al. [6]. The first video of a low-calorie yogurt assumes women concerned with physical attractiveness belong to women in a decorative role (hostile sexism). The second video of sausages assumes the role of housewife within the women’s category in a traditional role (benevolent sexism). The following are the external YouTube link for each video (in the Spanish language): 

Video 1: https://www.youtube.com/watch?v=fsUHteFjDMU (accessed on 1 December 2020).

Video 2: https://www.youtube.com/watch?v=6wIVxgT0Bug (accessed on 1 December 2020).

### 3.2. Measures

The questionnaire included three composites validated from previous studies: attitude toward advertisement (four items) by MacKenzie et al. [76]; ethical judgment (four items) by Brito-Rhor et al. [11]; and purchase intention (two items) by Zeng et al. [77]. All observable indicators were measured in a seven-point scale ranging from 7 (the best) to 1 (the worst), adapted to each item. Table 1 shows the composites and items. Composites have been validated by several academics.

### 3.3. Data Analysis

Partial least squares-structural equation modeling (PLS-SEM) was applied to analyze the hypothesized relations. SPSS (version 25.0, IBM Corp., Armonk, NY, USA) [78] and SmartPLS software (version Professional 3.3.3, SmartPLS GmbH, Schleswig, Germany) [79] computed the analyses. 

**Figure 1:**
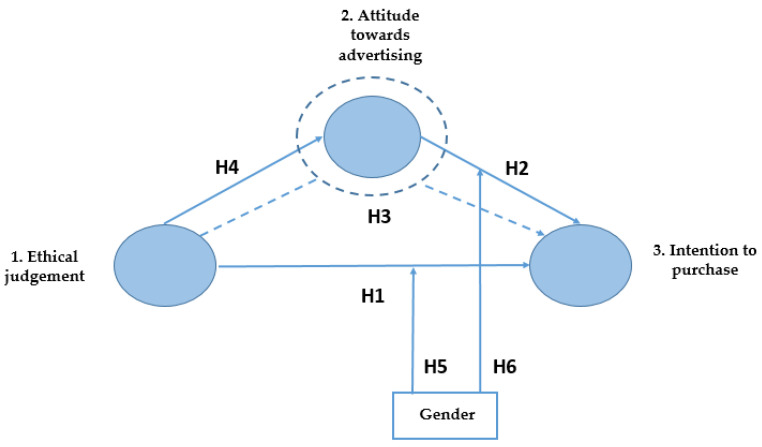
Research Model (videos 1 and 2). H means hypothesis. Source: Own elaboration.

Based on the hypotheses above-mentioned, we proposed a conceptual research model as illustrated in Figure 1, depicting the relationships, the direct impacts hypotheses, and the mediating variable, attitude toward advertising.

PLS-SEM does not require the normal distribution of the data. Nevertheless, highly non-normal data raise a problem in assessing the significances of the estimated values [80]. Suppose the absolute value of the index of skew is less or equal to 3.0 and the index of kurtosis is less or equal to 10.0, it can be said that the shape of the distribution may not be severely non-normal as Kline established [80], p. 77. However, according to Hair-Jr. et al. [75], the general guide states that distributions showing skewness or kurtosis indexes that exceed the absolute value of 1 are considered non-normal.

Following Hair et al. [81], the assessment of the PLS results includes the evaluation of the outer and the inner models. The assessment of the reflective measurement models includes indicator reliability, internal consistency reliability, convergent validity, and discriminant validity. Moreover, Heterotrait–Monotrait (HTMT) and Fornell and Lacker’s criterion ascertain discriminant validity (i.e., how different are the construct conceptually, composites in our case. Concerning the inner model, to determine redundancy in the inner model, the level of multicollinearity in the composites should be examined. Afterward, the significance and level of path coefficients and relevance of effects, the coefficient of determination, and the Stone-Geisser’s value Q^2^ were employed to assess the structural model.

As SmartPLS directly provides the variance inflation factors (VIF), we obtained these values to examine the degree of multicollinearity. Bootstrapping—with a configuration of 10,000 samples, percentile option, and one tail—was used to test the significance of path coefficients. Next, the dependent composite’s coefficient of determination (R-Square, R^2^) is the crucial criterion. It measures the variance of the endogenous composite caused by other exogenous composites in the inner model. Concretely, in consumer behavior, values of R^2^ of 0.20 are considered high [82], p. 14. Additionally, the endogenous composite’s cross-validated redundancy measure value Q^2^ should be higher than zero; this means that the exogenous latent variable exhibits predictive in-sample and out-of-sample relevance, in this case, configured with an omission distance of eight. Although it gives redundant information in the relevance of path coefficients, we used the Cohen’s effect size index (f^2^). This statistic quantifies the independent composite incremental explanation on the dependent composite. Thus, f^2^ values of 0.02, 0.15, and 0.35 mean that independent variables have a weak, medium, or substantial effect on the dependent not observed variables, respectively, according to Cohen ([83], pp. 413–414).

We followed the procedure established by Zhao et al. [84] concerning the mediation analysis. Furthermore, an additional and secondary criterion is the variance account for (VAF), which assesses the proportion of the indirect effect representing the total effect. In percentage, VAF below 20% is no mediation; VAF between 20% and 80% is partial mediation, and greater than 80% is complementary or competitive mediation, as described by Hair et al. [75], p. 225.

## 4. Results

### 4.1. Descriptive Analyses of the Observed Variables

Supplementary Table S1 displays the descriptive statistics of the observed variables. The requirements concerning the skewness and (excess) kurtosis were not satisfied in several variables. Nevertheless, this is not a severe non-normality based on the shape of the distribution.

### 4.2. Results of Hostile Sexism Video’s Model (Video 1) and Benevolent Sexism Video’s Model (Video 2)

#### 4.2.1. Measurement Models

Due to reasons of reliability and validity, it was necessary to recalculate the model in order to reach the minimum thresholds. The items not included were eliminated from the measurement model, thereby increasing the reliability and validity of the composites to achieve the correspondent requirements. Tables S2 and S3 display the results of these measurements.

Furthermore, the discriminant validity was evaluated through the application of three criteria. The Fornell and Lacker [85] criterion support the discriminant validity since the square root of the AVE of the construct is higher than the correlation between the other observed variables and composites (Table S4). According to HTMT criterion by Henseler et al. [86], all of the HTMT ratios are less than 0.90 (Table S4). Finally, the cross-loadings displays the maximum value of correlation between the composite and the corresponding indicators (Table S5). The results of the three criteria support the discriminant validity. 

In summary, we can conclude that the measurement model meets the required thresholds established by all of the above-mentioned criteria.

#### 4.2.2. Structural Models

Likewise, the measurement model achieved the minimum established thresholds provided by the literature. Thus, the coefficient of determination R^2^ evaluates the explanatory power of the model, in all cases in each model that are significant (R^2^ > 0.5) following Hair et al. [75]. Nowadays, some academics consider Q^2^ a between in-sample and out-of-sample predictive power measure since it is calculated based on omitting the in-sample values. The model fulfils the minimum required value for both models. Moreover, even R^2^ is substantial in all cases (see Tables S6 and S7). The f^2^ effect size is redundant to the path coefficients; however, as they are derived from R^2^, Tables S6 and S7 display their values. These last measures, f^2^, assess the strength of the inner relationships as the path coefficients do.

**Figure 2:**
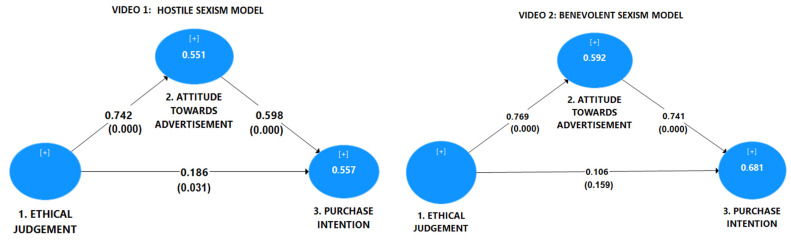
Estimated models (videos 1 and 2). Source: *p*-values into brackets, adapted from original figures obtained using [79].

Next, we will analyze the significance and relevance of the direct effects, indirect effects, and total effects. Figure 2 shows the estimated structural model for each video and the respective direct effects. Concerning the relevance of the path coefficients, on the one hand, the most relevant direct positive impact was the ethical judgment in explaining attitude toward advertisement (0.742 in the hostile video and 0.769 in the benevolent video). On the other hand, the following relevant coefficient (0.598 in video one and 0.741 in video two) showed the direct positive effect of attitude toward advertisement on purchase intention. In the hostile sexism model, the minor direct positive effect was the ethical judgment influence on purchase intention (0.186). Meanwhile, there was no direct effect in the benevolent sexism model. Therefore, hypothesis seven (H7) is supported. Table 2 display direct, indirect, total effect, type of mediation, and results of tests concerning video 1.

In addition, attitude toward advertisement mediates between ethical judgement and purchase intention, being a partial collaborative mediator between them in video 1; meanwhile, it was a full collaborative mediator between them in video 2. Additionally, the variance account for (VAF) was equal to the proportion of indirect effect from total effect (displayed in percentage in the Table). VAF less than 20% was considered no mediation; VAF between 20% and 80% was partial mediation and greater than 80% was full mediation. This secondary criterion concludes the same type of mediator for attitude toward advertising, partial, and full mediator, respectively, for video 1 and video 2. 

The decision about hypotheses, the different effects (direct, indirect, and total), whether these effects are significant or not, the type of mediation, and VAF concerning the video 2 are shown in Table 3, summarizing the comparisons under consideration. Hypotheses H2, H3, and H4 are supported in both models. H1 (1) (hostile sexism) is supported while H1 (2) (benevolent sexism) is rejected. Moderation analysis concerning Hypotheses H5 and H6 in both videos are rejected and is reported in Table 4. 

**Figure 3:**
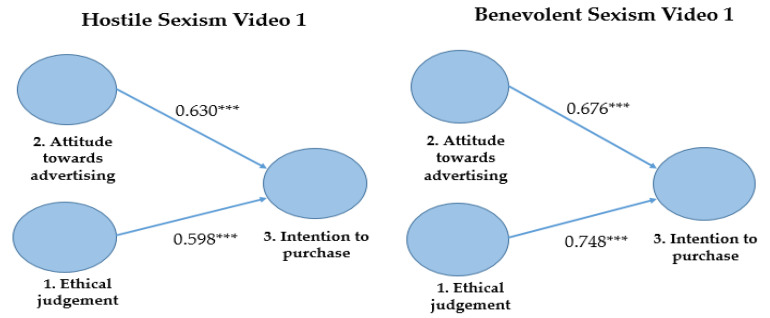
Total effects on Generation Z’s purchase intention of food products. Source: Own elaboration based on results from [79]. Note. *** *p* < 0.001.

Finally, Figure 3 displays the total effects. The most relevant in the hostile sexism model was the attitude toward advertising in terms of the total effect; meanwhile, ethical judgement was the most relevant in the benevolent sexism model.

## 5. Discussions and Conclusions

### 5.1. Theoretical Implications

This study showed some interesting findings that, from a theoretical perspective, can contribute to broadening the current knowledge of the perception of advertising gender stereotypes in the field of young people of Generation Z and their impact on the intention to buy food products. In particular, it is a novel study in the food industry since it has tested the perceptions of these stereotypes in this age cohort, which is so important in current and future food consumption. It is relevant to mention as new research proposals (i) the joint incorporation of ethical judgment and attitude toward advertising as antecedents of the intention to purchase food products as well as (ii) the study of the perceptions of Generation Z. 

Advertising using benevolent sexist stereotypes—housewife role—supports that ethical judgment (EJ) has no direct influence on purchase intention (PI). This result agrees with some previous authors [69,70]. However, in the case of hostile sexist advertising stereotypes—women concerned with physical attractiveness role—ethical judgment impacts directly and positively. Thus, our results agree with Zawisza et al. [12] concerning the United Kingdom, but differ from the results referring to Poland or South Africa, which showed an indirect relationship. The results also suggest that ethical judgment indirectly affects purchase intention through benevolent sexism or hostile sexism through advertising. This finding contributes to the knowledge of new antecedents of purchase intention in the food industry (attitude toward announcement and ethical judgement). Likewise, the present work allows us to differentiate the perception of benevolent and hostile sexism in young consumers and their emotional response, contributing new knowledge to research in business ethics.

In line with recent studies, which also use the PLS methodology [51,68], the attitude toward advertising (ATA) has a positive and direct impact on the purchase intention (PI). However, since there are no specific studies on the food industry or Generation Z, our findings fill some gaps in the literature. Similarly, the results show that the ethics of Gen Z on the intention to purchase food products when advertising uses stereotypes of benevolent sexism was not significant. This result is in contrast to Hernandez and Kaeck [47]. However, this relationship is direct and positive when advertising uses hostile sexist roles. In addition, the direct and positive relationship of ethical judgment (EJ) on attitude toward advertising (ATA) into the field of advertising of food products perceived by young people of Generation Z is another relevant finding [11,56].

### 5.2. Managerial Implications

The present research provides recommendations for professionals of food companies involved in the advertising communication of their products. Specifically, advertising managers in food and beverage companies could increment the purchase intention of young consumers focusing on the effects of the ethical judgment of the advertising. In the framework of hostile sexist stereotypes, ethical judgment has a direct positive impact on purchase intention. In the case of advertising with benevolent sexism stereotypes, an increment of this type of ethical judgment will not lead to a direct decrease in their desire to purchase the product, contrary to findings of previous studies [48,49,50,51]. However, ethical judgment indirectly affects purchase intention through the attitude toward the advertisement.

Previous studies have shown findings that hostile sexist female stereotypes negatively influence purchase intention [11,70]. For this reason, this type of female stereotype should be discouraged by advertising managers in food and beverage companies. In contrast, our study suggests that the purchase intention is not directly affected by the use of the housewife role since the relationship is indirectly positive. Therefore, it seems relevant to us to indicate that this relationship is different, depending on the analyzed country. In Spain, our results were similar compared with the United Kingdom report [12]. On the contrary, the ethical judgment did not directly influence purchase intention with the hostile woman role in the advertising in the case of Poland or South Africa [12]. These disparities in the results may imply that advertising companies use different communication strategies depending on the country in which they advertise.

The fact that young people belonging to Generation Z do not attach importance to benevolent sexist stereotypes through the role of women as housewives may have other implications in the workplace. As Singh and Banerjee [68] pointed out, after all, advertising is a way of influencing behavior and considerations of what is and is not considered right and wrong. Thus, as these young people enter the labor market and managerial positions, discriminatory work situations associated with a natural perception that the woman should take care of home and family may arise. Equally, this fact can generate aspirational conflicts for some women with the consequent loss of talent in organizations.

From an educational point of view, we also consider it advisable to emphasize gender equality education in schools and institutes, analyzing both hostile and benevolent sexist roles.

On the other hand, the finding of the direct influence of attitude toward advertising on purchase intention means that advertising companies need to take care of the attractiveness and interest of their advertisements to increase purchase intention.

Concerning the influence of ethical judgement on attitude toward advertisement, this study has shown that young people’s perception of the credibility and morality of advertising will directly affect product image and advertising interest. We therefore consider it essential that companies pay special attention to gender stereotypes in their advertising.

### 5.3. Limitations and Suggestions for Future Research

This research presents various limitations. The sample was an online convenience sample, so results could be biased to people who do not respond online. Additionally, the sample size was small, was carried out specifically on young people in Andalucía, Spain, and at a specific time over the year. Conducting analyses in Spain and other countries with larger samples could improve the knowledge and validate the results of this study. Moreover, it would be relevant to include attitude toward brand either as an antecedent or mediator between attitude toward advertisement and purchase intention. Finally, a comparison between the different generations, between Generation Z and Generation X or Y, will facilitate the target of the advertising strategies.

## Figures and Tables

**Table 1:** Composite (not observed variable) and measures (observed variables).

Composite (Latent Variables)	Measures (Observed Variables or Indicators)	Seven-Point Likert Scale	Label
1. Ethical Judgement	How do you see the performance of the characters?	1: Strongly incredible–7: Strongly credible	EJ1
Brito-Rhor et al. [11]	How do you feel about the use of images?	1: Strongly imprudent–7: Strongly prudent	EJ2
	How acceptable is it to you and your family?	1: Strongly unacceptable–7: Strongly acceptable	EJ3
	How does it seem to you morally?	1: Strongly immoral–7: Strongly moral	EJ4
2. Attitude toward Advertisement	Do you like the advertising?	1: Totally dislike–7: Totally like	ATA1
MacKenzie et al. [76]	What do you think about the advertising?	1: Not appealing–7: Very appealing	ATA2
	Are you interested in the advertising?	1: Not interesting–7: Very interesting	ATA3
	Do you think the advertising is offensive?	1: Not at all offensive–7: Very offensive	ATA4
3. Purchase Intention of Food Product	Do you want to buy the product after seeing the advertisement?	1: Not likely to buy–7: Very likely to buy	PI1
Zeng et al. [77]	Would I be able to purchase the product after seeing this ad?	1: Not likely to buy–7: Very likely to buy	PI2

Source: Own elaboration.

**Table 2:** Direct, indirect, total effect, type of mediation, and results of tests concerning video 1 (hostile sexism).

	Coefficient (BCa Bootstrap 95%CI)	Sig. *p*-Value	Hypotheses	Type Mediation and VAF
**Direct Path**				
H1 (1). Ethical judgment has a direct, positive influence on purchase intention (video 1).	0.186 (0.020; 0.346)	* (0.031)	Supported	-
H2 (1). Attitude towards advertisement has a direct, positive influence on purchase intention (video 1).	0.598 (0.465; 0.723)	*** (0.000)	Supported	-
H4 (1). Ethical judgment has a direct, positive influence on attitude towards advertisement (video 1).	0.742 (0.659; 0.806)	*** (0.000)	Supported	-
**Specific Indirect Path**				*-*
H3 (1). Ethical judgement indirectly affects purchase intention through attitude towards advertisement (video 1).	0.444 (0.353; 0.554)	*** (0.000)	Supported	VAF = 70.48% Partial collaborative mediation
**Total = Direct + Indirect**				*-*
H1 (1). Ethical judgment has a positive influence on purchase intention (video 1).	0.630 (0.510; 0.719)	*** (0.000)	Supported	-
H2 (1). Attitude towards advertisement has a positive influence on purchase intention (video 1).	0.598 (0.465; 0.723)	*** (0.000)	Supported	-
H4 (1). Ethical judgment has a direct, positive influence on attitude towards advertisement (video 1).	0.742 (0.659; 0.806)	*** (0.000)	Supported	-

Note: 10,000 subsamples. Bias-corrected and accelerated (BCa) bootstrap. Sig.: Significant, NS: Not significant. Variance account for (VAF). * *p* < 0.05; *** *p* < 0.001. Source: Own format of table elaboration with results obtained from [79].

**Table 3:** Direct, indirect, total effect, type of mediation, and results of tests concerning video 2 (benevolent sexism).

	Coefficient (BCa Bootstrap 95%CI)	Sig. *p*-Value	Hypothesis	Type Mediation and VAF
**Direct Path**				
H1 (2). Ethical judgment has a direct, positive influence on purchase intention (video 1).	0.106 (−0.061; 0.292)	NS (0.159)	Rejected	
H2 (2). Attitude towards advertisement has a direct, positive influence on purchase intention (video 1).	0.741 (0.564; 0.899)	*** (0.000)	Supported	
H4 (2). Ethical judgment has a direct, positive influence on attitude towards advertisement (video 1).	0.769 (0.682; 0.832)	*** (0.000)	Supported	
**Specific Indirect Path**				
H3 (2). Ethical judgement indirectly affects purchase intention through attitude towards advertisement (video 1).	0.570 (0.423; 0.736)	*** (0.000)	Supported	VAF = 84.32% Full collaborative mediation
**Total = Direct + Indirect**				
H1 (2). Ethical judgment has a positive influence on purchase intention (video 1).	0.676 (0.585; 0.753)	*** (0.000)	Supported	
H2 (2). Attitude towards advertisement has a positive influence on purchase intention (video 1).	0.741 (0.564; 0.899)	*** (0.000)	Supported	
H4 (2). Ethical judgment has a direct, positive influence on attitude towards advertisement (video 1).	0.769 (0.682; 0.832)	*** (0.000)	Supported	

Note: 10,000 subsamples. Bias-corrected and accelerated (BCa) bootstrap. Sig.: Significant, NS: Not significant. Variance account for (VAF). *** *p* < 0.001. Source: Own format table elaboration with results obtained from [79].

**Table 4:** Moderation analysis (video 1 and video 2).

Moderation	Coefficient (BCa Bootstrap 95%CI)	Sig. *p*-Value	Hypothesis
H5 (1). Gender moderates the relationship between attitude towards advertisement and purchase intention (video 1).	−0.000 (−0.103; 0.093)	NS (0.328)	Rejected
H6 (1). Gender moderates the relationship between ethical judgment and purchase intention (video 1).	0.031 (0.022; 0.255)	NS (0.497)	Rejected
H5 (2). Gender moderates the relationship between attitude towards advertisement and purchase intention (video 2).	−0.031 (−0.131; 0.053)	NS (0.288)	Rejected
H6 (2). Gender moderates the relationship between ethical judgment and purchase intention (video 2).	−0.046 (−0.144; 0.035)	NS (0.201)	Rejected

Note: Sig.: Significant, NS: Not significant. Source: Own format table elaboration with the results obtained from [79].

## Data Availability

Data available on request due to restrictions in privacy. The data are not publicly available due to further research.

## Supplementary Materials

The following supporting information can be downloaded at: https://www.mdpi.com/article/10.3390/foods11010053/s1, Table S1: Summary of descriptive analysis of observed variables; Table S2: Item reliability; Table S3: Item reliability summary, internal consistency reliability, and convergent validity; Table S4: Results for discriminant validity assessment; Table S5. Discriminant validity: Cross-loading criterion; Table S6: Measures of model’s predictive power and inner variance inflation factors concerning video 1; Table S7: Measures of model’s predictive power and inner variance inflation factors concerning video 2.
