# Supplementary material for: Generation Z Young People’s Perception of Sexist Female Stereotypes about the Product Advertising in the Food Industry: Influence on Their Purchase Intention
Source: Foods. 2021 Dec 27;11(1):53. doi: 10.3390/foods11010053 (PMC8750082; doi:10.3390/foods11010053)
Supplement: Supplementary file 1 [file foods-11-00053-s001.zip › foods-1507267-supplementary.pdf]

Article

GENERATION Z YOUNG PEOPLE'S PERCEPTION OF SEXIST FEMALE STEREOTYPES ABOUT THE PRODUCT ADVERTISING IN THE FOOD INDUSTRY: INFLUENCE ON THEIR PURCHASE INTENTION

Guillermo Bermúdez-González [1], Eva María Sánchez-Teba [2], María Dolores Benítez-Márquez [3,*], and Amanda Montiel-Chamizo [4]

[1,4] University of Malaga, 29071 Malaga, Spain; gjbermudez@uma.es
[2] University of Malaga, 29070 Malaga, Spain; emsanchezteba@uma.es
[3] University of Malaga, 29070 Malaga, Spain; bemarlo@uma.es
* Correspondence: bemarlo@uma.es

Abstract:

Previous studies have generated important insights into consumer behavior. However, no study has addressed how to persuade young people belonging to Generation Z to increase the purchase intention of food products from a gender perspective. Drawing on ambivalent sexism theory, this paper explores the influence of the attitude toward advertising and the ethical judgment to predict consumers' food product purchase intention. We applied a quantitative method, partial least squares structural equation modeling, to 105 individuals. Two advertisements with different food products and female role stereotype categories are using: (1) women in a traditional role or housewife's role (benevolent sexism), and (2) women in a decorative role or physical attractiveness (hostile). However, the results show that attitude toward advertising has a direct and positive influence on purchase intention in advertisement with benevolent sexism. In addition, the effect of ethical judgment on consumers' food product purchase intention is not significant. In the advertisement with hostile sexism, both—attitude toward advertising and ethical judgment—directly and positively impact purchase intention. The study provides a novelty conceptual model in the food industry for Generation Z and recommendations on the use of female sexist stereotypes in food and beverage advertising.

Keywords: Generation Z; advertising; gender stereotypes; ambivalent sexism; ethical judgment; attitude towards advertising; purchase intention; structural equation modeling; PLS-SEM.

Supplementary

Table S1. Summary of descriptive analysis of observed variables.

Label	Number	N	Min.	Max.	Mean	Std. Dev.	Skewness	Kurtosis
Observed Variable	Observed Variable	Stat.	Stat.	Stat.	Stat.	Statistic	Statistic	Statistic
Video 1. Ethical Judgment								
1.1.1_EJ1_V1	1	105	2.867	3	1	7	1.719	−0.348
1.1.2_EJ2_V1	2	105	3.762	4	1	7	1.509	−0.631
1.1.3_EJ3_V1	3	105	4.514	4	1	7	1.773	−1.051
1.1.4_EJ4_V1	4	105	3.61	3	1	7	1.807	−1.172
Video 1. Attitude Towards Advertisement								
1.2.1_ATA1_V1	5	105	3.61	4	1	7	1.715	−1.122
1.2.2_ATA2_V1	6	105	3.752	3	1	7	1.614	−0.832
1.2.3_ATA3_V1	7	105	3.524	3	1	7	1.668	−1.054
1.2.4_ATA4_V1	8	105	3.39	3	1	7	2.021	−1.382
Video 1. Purchase intention of food product								
1.3.1_PI1_V1	9	105	2.629	2	1	7	1.557	−0.51
1.3.2_PI2_V1	10	105	2.295	2	1	6	1.524	−0.508
Video 2. Ethical Judgment								
2.1.1_EJ1_V2	11	105	2.943	3	1	7	1.536	−0.647
2.1.2_EJ2_V2	12	105	4.038	4	1	7	1.696	−0.828
2.1.3_EJ3_V2	13	105	3.467	4	1	7	1.696	−0.669
2.1.4_EJ4_V2	14	105	3.086	3	1	7	1.708	−0.293
Video 2. Attitude Towards Advertisement								
2.2.1_ATA1_V2	15	105	2.933	3	1	7	1.545	−0.657
2.2.2_ATA2_V2	16	105	3.486	4	1	7	1.442	−0.523
2.2.3_ATA3_V2	17	105	2.924	3	1	7	1.739	−0.931
2.2.4_ATA4_V2	18	105	3.838	4	1	7	2.066	−1.216
Video 2. Purchase intention of food product								
2.3.1_PI1_V2	19	105	2.524	2	1	7	1.657	−0.416
2.3.2_PI2_V2	20	105	2.371	2	1	6	1.64	−0.434

Note: N. Sample Size. Min., Minimum; Max., Maximum. Stat., Statistic. Source: Own elaboration – [A1].

Table S2. Item reliability.

Composite/ Observed variables	Factor Loadings >0.7	Composite/ Observed variables	Factor Loadings >0.7
Video 1. Ethical Judgment		*Video 2. Ethical Judgment*	
1.1.1_EJ1_V1	-	2.1.1_EJ1_V2	-
1.1.2_EJ2_V1	0.895	2.1.2_EJ2_V2	0.913
1.1.3_EJ3_V1	-	2.1.3_EJ3_V2	0.899
1.1.4_EJ4_V1	0.926	2.1.4_EJ4_V2	0.939
Video 1. Attitude Towards Advertisement		*Video 2. Attitude Towards Advertisement*	
1.2.1_ATA1_V1	0.958	2.2.1_ATA1_V2	0.938
1.2.2_ATA2_V1	0.954	2.2.2_ATA2_V2	0.896
1.2.3_ATA3_V1	-	2.2.3_ATA3_V2	0.904
1.2.4_ATA4_V1	-	2.2.4_ATA4_V2	-
Video 1. Purchase Intention of Food Product		*Video 2. Purchase Intention of Food Product*	
1.3.1_PI1_V1	1	2.3.1_PI1_V2	1
1.3.2_PI2_V1	-	2.3.2_PI2_V2	-

Source: Own elaboration - [A1].

Table S3. Summary of factor loadings' confidence intervals, internal consistency reliability, and convergent validity.

Composites Observed variables	Factor Loadings >0.7 (Min.; Max.)	CA >0.7	RhoA >0.7	CR >0.7	AVE >0.5
Video 1. Ethical Judgment	(0.895; 0.926)	0.796*	0.811*	0.907*	0.830*
Video 1. Attitude Towards Advertisement	(0.954; 0.958)	0.906*	0.907*	0.955*	0.914*
Video 1. Purchase Intention of Food Product	1	1.000	1.000	1.000	1.000
Video 2. Ethical Judgment	(0.899; 0.939)				
Video 2. Attitude Towards Advertisement	(0.896; 0.904)				
Video 2. Purchase Intention of Food Product	1				

Note. CA: Cronbach's alpha [A2]; RhoA: Dijkstra–Henseler rho (also denotes by ρ_A) [A3]. CR: Composite reliability [A4]; AVE: Average variance extracted. Obtained from the 95% confidence intervals. Bootstrap's configuration: Bias-Corrected and Accelerated (BCa) Boostrap and one-tailed. Source: Own elaboration - [A1]

Table S4. Results for discriminant validity assessment: Fornell-Larcker and Heterotrait-Monotrait (HTMT) ratio

Fornell-Larcker and Heterotrait-Monotrait (HTMT) ratio						
	Video 1			Video 2		
	1. Ethical Judgment	2. Attitude Towards Advertisement	3. Purchase Intention of Food Product	1. Ethical Judgment	2. Attitude Towards Advertisement	3. Purchase Intention of Food Product
1. Ethical Judgment	0.911	0.871 (0.785; 0.946)	0.700 (0.572; 0.811)	0.917	0.851 (0.785; 0.946)	0.708 (0.785; 0.946)
2. Attitude Towards Advertisement	0.742	0.956	0.772 (0.713; 0.826)	0.769	0.913	0.862 (0.785; 0.946)
3. Purchase Intention of Food Product	0.630	0.736	1.000	0.676	0.822	1.000

Note. The square root of the average variance extracted (AVE) of each composite is in bold and located on diagonal. Over diagonal HTMT correlations ratios and under diagonal construct's inter-correlations. Bootstrap's configuration: 10,000 samples, Bias-Corrected and Accelerated (BCa) Boostrap and one-tailed. Source: Own elaboration - [A1].

Table S5. Discriminant validity: Cross-loading criterion.

Cross-loading criterion							
Video 1				Video 2			
Composites				Composites			
Indicators	1. Ethical Judgment	2. Attitude Towards Advertisement	3. Purchase Intention of Food Product	Indicators	1. Ethical Judgment	2. Attitude Towards Advertisement	3. Purchase Intention of Food Product
1.2_EJ2_V1	0.926	0.631	0.506	1.2_EJ2_V2	0.712	0.938	0.739
1.4_EJ4_V1	0.630	0.716	0.632	1.3_EJ3_V2	0.728	0.896	0.639
-	-	-	-	1.4_EJ4_V2	0.671	0.904	0.860
2.1_ATA1_V1	0.708	0.958	0.734	2.1_ATA1_V2	0.913	0.757	0.644
2.2_ATA2_V1	0.711	0.954	0.672	2.2_ATA2_V2	0.899	0.654	0.570
-	-	-	-	2.3_ATA3_V2	0.939	0.700	0.641
3.1_PI1_V1	0.630	0.736	1.000	3.1_PI1_V2	0.676	0.822	1.000

Note: The highest values expected are marked in bold. Source: Own elaboration - [A1].

Table S6. Video 1: Measures of model's predictive power and inner variance inflation factors.

Video 1	Inner Variance Inflation Factor VIF ⩽ 3.3		Explanatory Power		Predictive Power	Effect size f^2	
Dependent composites	*Constructs*		R^2 *Minimum cutoff* $R^2 \geq 0.1$ [A5]		Q^2 *(Predictive if* $Q^2 > 0$*)* [A6] [A7]	*Constructs*	
	1.	*2.*				*2.*	*3.*
1.1. Ethical Judgment	-		-	-	-	1,229 Substantial	0,035 Medium
1.2. Attitude Towards Advertisement	1	-	0.551*	Substantial [A8]	0.496	-	0.362 Substantial
1.3. Purchase Intention of Food Product	2.451	2.451	0.557*	Substantial [A8]	0.534	-	-

Note. 1. Ethical Judgment; 2. Attitude towards Advertisement; 3. Purchase Intention of Food Product. * $p < 0.05$. Test by bootstrapping 10,000 samples. Configuration: Bias-Corrected and Accelerated (BCa) Boostrap and one-tailed. Source: Own elaboration - [A1].

Table S7. Video 2: Measures of model's predictive power and inner variance inflation factors

Video 2	Inner Variance Inflation Factor VIF ⩽ 3.3		Explanatory Power		Predictive Power	Effect size f^2	
Dependent composites	*Constructs*		R^2 *Minimum cutoff* $R^2 \geq 0.1$ [A5]		Q^2 *(Predictive if* $Q^2 > 0$*)* [A6] [A7]	*Constructs*	
	1.	*2.*				*2.*	*3.*
2.1. Ethical Judgment	1.000	2.451	-	-	-	1.451 Substantial [A5]	0.014 Less than weak [A5]
2.2. Attitude Towards Advertisement	-	2.451	0.592*	Moderate [A3]	0.492	-	0.701 Substantial [A5]
2.3. Purchase Intention of Food Product	-	-	0.681*	Moderate [A3]	0.655	-	-

Note. 1. Ethical Judgment; 2. Attitude towards Advertisement; 3. Purchase Intention of Food Product. * $p < 0.05$. Test by bootstrapping 10,000 samples. Configuration: Bias-Corrected and Accelerated (BCa) Boostrap and one-tailed. Source: Own elaboration - [A1].

Supplementary materials' references

A1. Ringle CM, Wende S, Becker JM. Software "SmartPLS 3" (version Professional 3.3.5, update 2021). Boenningstedt; 2015. Available from: http://www.smartpls.com (last accessed on 26th December 2021).

A2. Cronbach LJ. Coefficient alpha and internal structure of test. *Psychometrika.* **1951**; *16*: p. 297–334.

A3. Dijkstra TK, Henseler J. Consistent partial least squares path modeling. *MIS Quarterly*. **2015**; *39*(2): p. 297–316.

A4. Werts CE, Linn RL, Jöreskog KG. Intraclass reliability estimate: Testing structural assumption. *Educ. Psychol. Meas.* **1974**; *34*: p. 25–33.

A5. Falk RF, Miller NB. *A primer for soft modeling* Akron: University of Akron Press; 1992.

A6. Stone M. Cross-validatory choice and assessment of statistical predictions. *J. R. Stat. Soc., B: Stat. Soc. (Methodological).* **1974**; *36*(2): p. 111–147.

A7. Geisser S. The predictive sample reuse method with applications. *J. Am. Stat. Assoc.* **1975**; 70(350): p. 320–328.

A8. Hair-Jr JF, Hult GTM, Ringle CM, Sarstedt M. *A Primer on Partial Least Squares Equation Modeling (PLS-SEM).* 1st ed. Thousand Oaks, California (United States): Sage Publication, Inc.; 2014.

A9. Hair-Jr JF, Hult GT, Ringle CM, Sarstedt M, Castillo-Apraiz J, Cepeda Carrion G, et al. *Manual de Partial Least Squares Structural Equation Modeling (PLS-SEM).* 2nd ed. Barcelona: OmniaScience (Omnia Publisher SL); 2019.
